# Altered Functional Mitochondrial Protein Levels in Plasma Neuron-Derived Extracellular Vesicles of Patients With Gadolinium Deposition

**DOI:** 10.3389/ftox.2021.797496

**Published:** 2022-01-12

**Authors:** Edward J. Goetzl, Holden T. Maecker, Yael Rosenberg-Hasson, Lorrin M. Koran

**Affiliations:** ^1^ School of Medicine, University of California San Francisco, San Francisco, CA, United States; ^2^ Human Immune Monitoring Center, Microbiology and Immunology, Stanford University Medical Center, Stanford, CA, United States; ^3^ Department of Psychiatry and Behavioral Sciences, Stanford University Medical Center, Stanford, CA, United States

**Keywords:** exosomes, toxic encephalopathy, mitochondrial functions, gadolinium deposition disease, GBCA

## Abstract

The retention of the heavy metal, gadolinium, after a Gadolinium-Based Contrast Agent-assisted MRI may lead to a symptom cluster termed Gadolinium Deposition Disease. Little is known of the disorder’s underlying pathophysiology, but a recent study reported abnormally elevated serum levels of pro-inflammatory cytokines compared to normal controls. As a calcium channel blocker in cellular plasma and mitochondrial membranes, gadolinium also interferes with mitochondrial function. We applied to sera from nine Gadolinium Deposition Disease and two Gadolinium Storage Condition patients newly developed methods allowing isolation of plasma neuron-derived extracellular vesicles that contain reproducibly quantifiable levels of mitochondrial proteins of all major classes. Patients’ levels of five mitochondrial functional proteins were statistically significantly lower and of two significantly higher than the levels in normal controls. The patterns of differences between study patients and controls for mitochondrial dynamics and mitochondrial proteins encompassing neuronal energy generation, metabolic regulation, ion fluxes, and survival differed from those seen for patients with first episode psychosis and those with Major Depressive Disorder compared to their controls. These findings suggest that mitochondrial dysfunction due to retained gadolinium may play a role in causing Gadolinium Deposition Disease. Larger samples of both GDD and GSC patients are needed to allow not only testing the repeatability of our findings, but also investigation of relationships of specific mitochondrial protein deficiencies or excesses and concurrent cytokine, genetic, or other factors to GDD’s neurological and cognitive symptoms. Studies of neuronal mitochondrial proteins as diagnostic markers or indicators of treatment effectiveness are also warranted.

## Introduction

The retention of the heavy metal gadolinium (Gd) after an MRI with a Gadolinium-Based Contrast Agent may lead to a symptom cluster termed Gadolinium Deposition Disease (GDD) that includes neuropathic pain, new onset frequent headaches, muscle fatigue, and cognitive complaints (Semelka et al., 2016a). Little is known of the pathophysiology underlying GDD, but a recent study reported abnormally elevated serum levels of pro-inflammatory cytokines compared to normal controls (Maecker et al., 2020). A second study of GDD patients reported abnormally elevated or reduced levels of serum cytokines during the 24 h after DTPA chelation to remove Gd (Maecker et al., 2021). Gd enhancement of cytokine levels has also been observed in animal models and cell cultures.

Gd also interferes with mitochondrial function through its action as a calcium channel blocker at both the cellular and the mitochondrial membranes (Rogosnitzky and Branch, 2016). Gd^3+^ increased mitochondrial membrane fluidity, decreased membrane potential, and increased cytochrome c release in rodent liver cells and isolated mitochondria, suggesting ionic Gd can enter cells and bind to mitochondria. (Liu et al., 2003; Zhao et al., 2014). In an *in vitro* study of cultured rat brain cortical neurons, GdCl_3_ inhibited mitochondrial function, including decreasing ATP synthesis, and increased cell death (Feng et al., 2010). Also *in vitro*, clinically relevant doses of GBCAs have a toxic effect on mitochondrial respiratory function and on the viability of human basal ganglia neurons (Bower et al., 2019).

Pain with neuropathic quality; new onset, frequent headaches; cognitive difficulties (“brain fog”); and muscle twitching are very common GDD symptoms (Ramalho et al., 2017; Maecker et al., 2020). Although GBCAs and Gd are retained in human brain (Levine et al., 2018; Radbruch et al., 2020) note that there is no evidence that these retained molecules cause histological changes or clinical symptoms. In animal studies, however, linear GBCAs induce pain hypersensitivity and small fiber neuropathy (Alkhunizi et al., 2020; Radbruch et al., 2020).

Newly developed methods allow isolation of human plasma neuron-derived extracellular vesicles (NDEVs) containing reproducibly quantifiable levels of mitochondrial proteins of all major classes. Specific profiles of abnormal levels have been observed in first episode psychosis (FEP) (Goetzl et al., 2020) and major depressive disorder (MDD) (Goetzl et al., 2021). In view of retention of the heavy metal Gd in GDD patients, their frequent complaints of neuropathic pain, “brain fog,” new onset, frequent headaches, and muscle fatigue, and the animal and *in vitro* studies cited above, we undertook the current study of exosomes reflecting brain mitochondrial function to investigate the possibility that impaired mitochondrial function could be related these symptoms.

## Methods

### Patients

Eleven study participants were enrolled from March 2019 to January 2020 at a Chapel Hill, NC medical clinic where they had come for Ca-DTPA chelation to remove Gd. All signed a Stanford University Medical Center IRB-approved Informed Consent for use of their blood samples and data. Since the benefit or lack of benefit of off-label Ca-DTPA chelation was not under study, consent for this treatment was given to the treating physician as part of ordinary medical practice and was not part of the Stanford IRB form. All diagnoses were made by the treating physician, who is an author of the first peer-reviewed articles describing GDD (Semelka et al., 2016a) and Gadolinium Storage Condition (GSC) (Semelka et al., 2016b). The nine GDD patients met the condition’s diagnostic criteria: normal or near normal kidney function; an unprovoked 24-h Gd urine excretion level exceeding the laboratory norm ≥28 days after the symptom-inducing MRI; and, new onset within 28 days of the GABA-assisted MRI of ≥3 of 8 symptoms: cognitive disturbance, extremity pain, arthralgia, chest wall pain, skin pain, headache, skin induration, and skin hyperpigmentation. The patients were aged >18 years; had no neurological or psychiatric disorder; no medical condition or current medication known to strongly influence serum cytokine levels, e.g., an infectious disease, cancer, rheumatoid arthritis, or an autoimmune disorder; and, no prior treatment for GDD with chelation or immune system modulation (e.g., steroid use) (Table 1).

**TABLE 1:** Patient clinical data.

Patient	Age	Sex	Pre- chelation Gd (mcg/24 h)	Post-chelation Gd (mcg/24 h)	# Of GBCA MRIs	Time since last GBCA dose	Time since symptom onset
AX	49	M	0.7	25	2	4 months	6 months
BG^a^	59	M	0.5	23	>15	4 years	no sx
KGB	48	F	0.7	34	17	2 years	10 years
CMB	44	F	0.2	4.7	3	1 year	1 year
SHW	55	F	0.4	25	7	2 years	7 years
NRQ	60	F	0.5	8.7	1^b^	4 months	4 months
RTC^a^	59	M	0.5	31	>15	4.25 years	no sx
LSB	66	M	0.2	17	3^b^	2 years	7 years
TBC	59	F	0.9	29	5	4 months	4 years
DCC	59	M	<0.1	5.7	2^b^	4 years	14 years
DAP^a^	66	M	<0.1	15	2	6 months	no sx
KGT	48	F	0.4	14	17	2.25 years	10 years
SBO	57	F	1.4	4.9	4	3 months	1 year

Abbreviations: Gd = gadolinium; mcg/24 h = micrograms/24 h; GBCA, gadolinium based contrast agent; MRI, magnetic resonance imaging; mo = month; yr = year; sx = symptoms.

a GSC, patient.

b Patient received only one GBCA, brand.

Note: BG, and RTC, are the same patient at different time points, as are KGB, and KGT.

Two patients with GSC, in which gadolinium has been retrained without accompanying symptoms (Semelka et al., 2016b), were also included. They came to the clinic concerned about Gd retention from their MRIs and requested DTPA chelation treatment to remove it. Control group participants, matched by age and sex to study patients, had no radiological procedures within 5 years and no exposure to Gd.

### Laboratory Methods

The 24-h urine specimens for Gd analysis were collected the 24 h before and during approximately 24 h after Ca-DTPA chelation. Doctor’s Data, Inc. (DDI) determined the Gd amounts via inductively coupled plasma mass spectrometry (See: https://doctorsdata.com/licensing). The DDI upper limit of normal (95th percentile) is ≤0.6 μg/24 h for women, and ≤1.0 μg/24 h for men, based on unprovoked urine samples from 336 women and 204 men asked to refrain from a GBCA-enhanced MRI for ≥48 h before starting urine collection.

To obtain plasma samples, 8 ml of blood was drawn at the start of Ca-DTPA chelation therapy, the serum separated in standard fashion, and the collection tubes put in dry ice and shipped to the Stanford Human Immune Monitoring Center laboratory, where they were stored frozen until the analyses for mitochondrial protein determination was performed. Patients had discontinued all medications and over-the-counter supplements for 3 days before the urine and blood samples were obtained.

The methods utilized to obtain the mitochondrial proteins from the plasma exosomes are described in detail elsewhere (Goetzl et al., 2021). Neuron-derived extracellular vesicles (NDEVs), including exosomes, were harvested from 0.25 ml of plasma of GDD and GSC patients and control participants by sequential ExoQuick precipitation and anti-L1CAM antibody immunoabsorption. The mean NDEV counts and sizes, as well as CD81 exosome marker levels, were the same in patients and controls. NDEV proteins were quantified using enzyme-linked immunosorbent assay (ELISA) kits. The ELISA quantifications were performed blind to whether they were from control subjects or patients and blind to all patient data.

In comparing the mean mitochondrial protein levels between GDD/GSC patients and normal controls, p-values were calculated using a two-sample version of Student’s t test (GraphPad, San Diego, CA).

## Results

Table 1 displays the 11 study patients’ demographic characteristics, 24-h urine Gd amounts on day before and the day after chelation, number of GBCA MRIs received, time between the last GBCA dose and the study blood draw, and time since GDD symptom onset. One GDD patient (BG-RTC) and one GSC patient (KGB-KGT) each provided serum samples on two occasions 3 months apart.

The reasons for patients’ MRIs were back pain (3), abdominal pain (2), throat pain (1), post-hysterectomy scan (1), breast CA (absent, 1), prostate CA (absent, 1), enlarged liver (1), and headache (1).

No clear relationship was seen between the pre- or post-chelation 24-h urine amounts and the time since the most recent GBCA-assisted MRI.

Of the twelve proteins within the four mitochondrial functional classes, plasma levels of five proteins were statistically significantly lower and of two higher in patients than in controls. Plasma levels of the remaining five proteins showed no statistically significant difference between GDD/GSC patients and controls (Table 2).

**TABLE 2:** CD81-normalized mitochondrial protein levels of GDD/GSC patients and controls.

Mitochondrial protein	Control levels Mean ± SEM	GDD/GSC patient levels Mean ± SEM	*p*-value	GDD/GSC patients’ levels compared to control levels	Comparison of GDD/GSC patients to controls vs Comparison of FEP and MDD patients to their controls
*Dynamic and Maintenance Functions*					
TFAM	979 ± 67.8	1,050 ± 78.7	0.5019	NS	Same
SNPH	1,070 ± 707	1,093 ± 108	0.2888	NS	Unalike
MY06	12,883 ± 522	25,345 ± 1935	0.0001	↑ 197%	Unalike
LETM1	2,295 ± 178	1,072 ± 136	<0.0001	↓ 47%	Same
*Energy Generation*					
NMNAT2	14,918 ± 1,297	8,710 ± 1,300	0.0033	↓ 58%	Same
SARM1	1,045 ± 58.8	1,470 ± 109	0.005	↑ 141%	Overlap
Complex 1–6	1,203 ± 175	1,108 ± 203	0.726	NS	Unalike
Complex III-10	669 ± 54.5	685 ± 51.6	0.8411	NS	Unalike
*Metabolic Regulation and Cellular Survival*					
Humanin	1,055 ± 76.8	624 ± 60.9	0.00002	↓ 59%	Same
MOTS-c	127,773 ± 995	109,727 ± 6,543	0.1189	NS	Unalike
*Mitochondrial Biogenesis*					
PGC1α	875 ± 62.6	364 ± 26.0	<0.0001	↓ 42%	Unalike
NRF2	1,421 ± 114	833 ± 64.9	0.0001	↓ 59%	Same

Fifth column: the mean percentage difference between GDD/GSC, patients’ levels and control levels; NS, no statistically significant difference. Sixth column: Same = the direction and approximate magnitude of GDD/GSC patient differences from controls are the same as those for FEP (First Episode Psychosis) and MDD (Major Depressive Disorder) patients vs their controls; Unalike = distinct differences in comparisons to controls than were observed for FEP and MDD patients compared to their controls; Overlap = similarity to differences from controls for only FEP or MDD patients, not both patient groups. Abbreviations**:** Complex I-6 = subunit 6 of NADH-ubiquinone oxidoreductase; complex III-10, subunit 10 of cytochrome b-c1 oxidase; NRF2 = type 2 nuclear respiratory factor; MY06 = myosin VI; PGC-1α = PPAR γ, coactivator-1α; MOTS-c, mitochondrial open-reading frame of the 12S rRNA-c; NMNAT2 = nicotinamide mononucleotide adenylyl transferase 2; SNPH, syntaphilin; LETM1 = leucine zipper EF-hand containing transmembrane 1 protein; SARM-1, Sterile Alpha and TIR, motif-containing protein 1; and TFAM, transcription factor A mitochondrial.

For five proteins, the direction and approximate magnitude of the differences in GDD/GSC patients’ plasma level versus the levels in controls were the same as for comparisons in earlier studies between FEP patients (Goetzl et al., 2020) and MDD patients (Goetzl et al., 2021) and their respective controls. For six proteins, the differences between GDD/GSC patients and controls were distinctive and unlike those noted in the comparisons done in the studies of FEP and MDD patients. The GDD/GSC differential increase in SARM1 resembled the differential increase seen in MDD patients, but FEP patients exhibited no significant difference from their controls.

## Discussion

Our findings of abnormal function in brain-derived mitochondria of GDD patients are the first reports utilizing plasma exosome analysis. They suggest that mitochondrial dysfunction may be involved in the pathophysiology of the neuropathic pain, “brain fog,” and muscle fatigue reported by GDD patients (Semelka et al., 2016a; Maecker et al., 2020).

The fact that similar mitochondrial changes were found in the two GSC patients, who lacked these symptoms, suggests that mitochondrial dysfunction is not a sufficient cause. It may be unrelated, but more probably, is a necessary or contributing cause. Mitochondrial dysfunction may join with Gd’s generation of abnormally elevated levels of specific cytokines as compared to levels in both normal controls (Maecker et al., 2020) and a small group of GSC patients (Maecker et al., 2021). Some of these cytokines, particularly TNF, IL-6, and MCP3, affect pain sensation. Other contributing causes may lie in genetic differences between GDD patients and both GSC patients and normal controls. Genetic contributions to disease occurrence are common in human disorders as diverse as bipolar disorder and breast cancer, along with thousands of rare diseases (Mistry et al., 2018; Wise et al., 2019; Escala-Garcia et al., 2020). The interplay between effects of genetics, immunology, and environmental exposure on cell biochemistry in non-alcoholic steatohepatitis may be analogous to the interplay of factors underlying the infrequent occurrence of GDD in individuals exposed to the heavy metal Gd via a GBCA-assisted MRI (Wree et al., 2013).

Results for individual mitochondrial proteins suggest a variety of possible pathophysiological pathways to GDD symptom formation. NDEV levels of MY06 were higher in GDD/GSC patients than in controls. MY06 is an unconventional myosin responsible for ruffled neuronal membrane structure and the tethering of mitochondria and some membrane vesicles to neuronal presynaptic microfilaments. The increased MY06 levels in plasma NDEVs of GDD/GSC subjects suggests that adherence of their mitochondria to neuronal microfilaments is altered, with consequently increased turnover of neuronal presynaptic but not of axonal mitochondria since NDEV levels of SNPH are normal (Table 2). By ATP provision and Ca++ buffering, the presynaptic mitochondria play a critical role in synaptic development, functioning, and pruning, The significance in GDD of this increased turnover is not clear, although the trafficking and function of presynaptic mitochondria in general are areas of active research (Devine and Kittler, 2018).

Inner mitochondrial membrane levels of LETM-1, a calcium channel/calcium channel enhancer of unclear mechanisms involved in calcium homeostasis, were significantly lower in GDD/GSC patients than controls. This may be attributable to an effect of Gd on the LETM-1 role in Ca^++^ fluxes directly or indirectly by influencing LETM-1 actions in maintaining mitochondrial membrane structure and respiratory functions (Natarajan et al., 2021). Ca++ fluxes across cellular and mitochondrial membranes are critical to a large number of physiological products including mitochondrial energy production (Feng et al., 2010) and to muscle function and fatigue (Eshima et al., 2014). If and how changes in particular Ca++ fluxes and impaired mitochondrial energy production might relate to GDD symptoms such as cognitive impairment and muscle fatigue can only be elucidated by future studies.

Levels of NMNAT2, the enzyme involved in generating nicotinamide adenine dinucleotide (NAD), were significantly lower in the patients. NMNAT2 (along with SARM1) is vital for mitochondrial energy generation via establishing mitochondrial concentrations of NADH and NAD+, which are as important in energy generation as the NADH/NAD+ ratio (Stein and Imai, 2012). SARM1 is involved in cellular NAD degradation. Thus, the lower NMNAT2 and higher SARM1 levels suggest that mitochondrial NADH plus NAD+ levels would be lower and energy production reduced in GDD/GSC patients. This speculation, however, requires measuring energy production in unextracted NDEVs and intact mitochondria.

Levels of the metabolic regulatory peptide Humanin were also significantly lower. Humanin helps protect neurons from stressors, restrains apoptosis, reduces neuroinflammation, preserves synaptic proteins, and assists in glucose metabolism. Reduced levels suggest that retained Gd lowers mitochondrial resistance to adverse stressors, including possibly the increased levels of pro-inflammatory cytokines reported in GDD (Maecker et al., 2020). Again, confirmation in unextracted NDEVs and intact mitochondria is required.

PGC1alpha and NRF2 both promote mitochondrial DNA replication, termed biogenesis, and several functional activities (Gureev et al., 2019). Their levels were significantly lower in GDD/GSC patients than in controls, with consequent depression of mitochondrial biogenesis and generation of ATP and ROS (Kelly and Scarpulla, 2004).

The lower levels of NMNAT2, Humanin, PGC1alpha and NRF2, and the resultant impairment in mitochondrial energy production, and the decreased restraint of neuronal apoptosis, protection from neuroinflammation, and regulation of ROS have plausible physiological pathway connections to the GDD complaints of neuropathic pain (Sui et al., 2013) and cognitive impairment (“brain fog”) (Han et al., 2020). Muscle fatigue is a very common complaint in primary mitochondrial diseases (Zolkipli-Cunningham et al., 2018) and mitochondrial energy production is central to muscle function. How impaired mitochondrial function might lead to new onset, frequent headaches is not clear.

The GDD patients’ post-chelation 24-h urine Gd amounts were 6–59 times the DDI laboratory norm for individuals not exposed to GBCAs. This suggests that the amount of retained Gd needed to contribute to evoking GDD symptoms in susceptible individuals may be lower than these multiples. The absence of a relationship between pre- and post-chelation 24-h urine Gd amounts may reflect differences in the number of MRIs received and in the stability constants and excretion patterns of the different GBCAs utilized (Lancelot, 2016; Ramalho et al., 2016). The time from the most recent MRI and from GDD symptom onset varied widely among patients. This variation does not, however, diminish the validity of the mitochondrial protein results. GDD is a chronic illness and the GDD patients were continuously symptomatic. In other chronic conditions, length of illness does not invalidate laboratory findings in symptomatic patients, e.g., elevated serum glucose in diabetes, presence of Mycobacterium tuberculosis in tuberculosis patients’ sputum, elevated BUN in chronic renal failure. Moreover, as noted, the GDD and GSC patients excreted 24-h urine Gd amounts post-chelation that are many times greater than the DDI, Inc. laboratory norm.

The pattern of differences between GDD/GSC patients and normal controls for twelve mitochondrial proteins encompassing neuronal energy generation, metabolic regulation, and ion fluxes were distinctive, i.e., these patterns differed from the patterns of differences seen for FEP (Goetzl et al., 2020) and MDD patients (Goetzl et al., 2021) and their controls. This suggests that in GDD patients, retained Gd (or retained GBCA) has effects on mitochondrial function distinct from those related to these other disorders. Nonetheless, the presence of signs of brain mitochondrial dysfunction in three disorders in which mental symptoms are prominent indicates the need for rapidly mounting studies to clarify specific relationships of mitochondrial functional markers to specific symptoms, to symptom severity, and to results of therapeutic trials.

This study is limited by small sample size and by the absence of independent rater quantitative ratings of the severity of patients’ symptoms. Future studies should utilize validated symptom rating scales to obtain these measures. Larger samples of both GDD and GSC patients are needed to allow not only testing the repeatability of our findings, but also to allow identification of additional factors involved in the development of GDD symptoms. Larger studies may elucidate relationships of specific mitochondrial protein deficiencies or excesses and concurrent cytokine, genetic, or other factors to the neurological, cognitive and skeletal muscle symptoms of GDD.

The pathophysiology of GDD symptoms remains to be definitively established. Still, the mitochondrial functional abnormalities reported here, as well as the reported cytokine abnormalities, deserve further exploration and elaboration. Mitochondrial function markers might prove useful for diagnosis or as markers of treatment effectiveness. If mitochondrial dysfunction is proven to be involved in causing GDD symptoms, studies in GDD of treatments that lessen this dysfunction in other disorders will deserve pursuit.

## Data Availability

The raw data supporting the conclusions of this article will be made available by the authors, without undue reservation.

## Abbreviations

GBCA, Gadolinium Based Contrast Agent; Gd, gadolinium; GDD, Gadolinium Deposition Disease; GSC, Gadolinium Storage Condition; FEP, First Episode Psychosis; MDD, Major Depressive Disorder; NDEV, Neuron-derived extracellular vesicle.
